# SARS-CoV-2 Seroprevalence in Indoor House Cats From the Lisbon Area During the COVID-19 Pandemic, 2019–2021

**DOI:** 10.1155/tbed/1543922

**Published:** 2024-11-21

**Authors:** Isa Moutinho, Mafalda Henriques, Sara Cardoso, Teresa da Penha Coutinho, Carlos Penha-Gonçalves, Jocelyne Demengeot, Miguel Castanho, Luís Tavares, Solange Gil, Telmo Nunes, Frederico Aires-da-Silva

**Affiliations:** ^1^Center for Interdisciplinary Research in Animal Health (CIISA), Faculty of Veterinary Medicine, University of Lisbon, Lisbon, Portugal; ^2^Associate Laboratory for Animal and Veterinary Sciences (AL4AnimalS), Lisbon, Portugal; ^3^Instituto Gulbenkian de Ciência, Oeiras, Portugal; ^4^Instituto de Medicina Molecular-João Lobo Antunes, Faculdade de Medicina, Universidade de Lisboa, Lisboa, Portugal

## Abstract

The susceptibility of various animal species to severe acute respiratory syndrome coronavirus 2 (SARS-CoV-2) infection has been studied extensively. Cats have garnered significant concern due to their high susceptibility and proximity to humans. This study aimed to evaluate the susceptibility and antibody response in house cats exposed to SARS-CoV-2 when human infection was spreading in the Lisbon area during the 2019–2021 period. A total of 733 serum samples were collected and characterized from cats admitted to the Veterinary Teaching Hospital of the Faculty of Veterinary Medicine at the University of Lisbon (HEV-FMV-ULisboa). All samples were tested by enzyme-linked immunosorbent assay (ELISA) for immunoglobulin M (IgM) and immunoglobulin G (IgG) anti-SARS-CoV-2 whole Spike and receptor-binding domain (RBD) proteins from the Wuhan-Hu-1 isolate and 14.7% (108/733) tested positive, suggesting exposure to the human virus. Surrogate virus neutralization test (sVNT) against the Wuhan-Hu-1 isolate showed that 20.4% of ELISA positive samples (22/108) harbored neutralizing antibodies against the virus. The 22 most promising serum samples were retested using ELISA and sVNT against Alpha, Delta, and Omicron SARS-CoV-2 variants. Notably, these samples exhibited antibodies that were capable of recognizing and neutralizing these variants. Subsequent neutralization assays confirmed that the serum samples effectively inhibited the infection process of Wuhan-Hu-1 D614G, Delta, and Omicron SARS-CoV-2 pseudotyped viruses. Our findings indicate that cats were exposed to SARS-CoV-2 infection during the pandemic period and generated highly effective and broadly neutralizing antibodies against the virus. Although cats have not been demonstrated to significantly contribute to the spread of SARS-CoV-2, their high susceptibility to asymptomatic infection underscores the importance of investment in preventive surveillance measures. In summary, our study reinforces the notion that cats naturally infected with SARS-CoV-2 represent a valuable anthroponotic disease model in house settings and might be a potential source for the development of antibodies against SARS-CoV-2 in tackling future outbreaks with a One Heath perspective.

## 1. Introduction

The past two decades have been marked by significant disease outbreaks associated with the emergence of new coronaviruses. First, severe acute respiratory syndrome (SARS) occurred in 2002, followed by Middle East respiratory syndrome (MERS) in 2012 [1–3]. In 2019, a new coronavirus associated with high incidence of respiratory diseases was identified and named severe acute respiratory syndrome coronavirus 2 (SARS-CoV-2) [4]. SARS-CoV-2 and the resulting disease, COVID-19, quickly spread worldwide, becoming one of the largest public health threats ever recorded [5]. Similar to other coronaviruses that cause human diseases in the past, SARS-CoV-2 is thought to have a zoonotic origin, presumably in RaBtCoV/4991/RaTG13 (RATG13), a virus found in *Rhinolophus* bats that may have reached humans through other animal reservoirs [4, 6]. Human-to-human transmission is the main driver of COVID-19 spreading; however, SARS-CoV- 2 infection has been reported in domestic, farm, wild, and captive animals that are in close contact with infected humans [7].

The first human-to-animal spillover events were reported in 2020 in mink farms in the Netherlands and Denmark. Minks were found to have respiratory disease, increased mortality, and an epidemiological link was established using viral RNA sequences isolated from these animals and farm workers [8–10]. Captive zoo felines including lions, tigers, cougars, and snow leopards have also been shown to be susceptible to SARS-CoV-2. These animals presented lethargy, cough, and nasal discharge with the detection of viral RNA in the upper respiratory tract and feces [11–14]. SARS-CoV-2 infections have also been reported in wild animals. In a survey conducted on a white-tailed deer population, the SARS-CoV-2 Omicron variant was detected in deer nasal swabs and serum samples tested positive for SARS-CoV-2 specific antibodies [15, 16]. Nevertheless, the greatest concern to human health is related to companion animals, such as dogs and cats, owing to cohabitation with these animals and their close contact with their owners. The SARS-CoV-2 susceptibility in dogs and cats has been confirmed through the detection of viral RNA in nasal and fecal swabs, as well as through seroprevalence surveys, indicating the development of antibodies against the virus [17–28].

The cellular receptor angiotensin-converting enzyme II (ACE2) plays a key role in the infection process, mediating the virus entry into the target cell. Some studies have shown that differences in ACE2 sequences are responsible for the variable susceptibility of different species to SARS-CoV-2 infection [29]. According to recent studies, despite the high sequence homology between human and canine ACE2, dogs appear to be less susceptible to infection, probably due to the lower expression of these cell receptors in their respiratory tracts [30, 31]. In contrast to dogs, cats have been found to be highly susceptible to SARS-CoV-2 infection, with several studies indicating that they can spread the virus orally, nasally, or through feces [32, 33]. Generally, cats remain asymptomatic or show mild symptoms, such as coughing or sneezing, and can transmit the virus to other cats via aerosols [17, 34–36]. The feline ACE2 cell receptor is one of the most similar receptors to its human counterpart, explaining the high susceptibility of these animals to SARS-CoV-2 infection [37].

Owing to their close and frequent contact with humans and other domesticated species, several studies have been conducted to determine whether cats play an active role in disease spreading [17, 19–28, 32–36, 38]. In the present study, we aimed to evaluate the exposure and antibody response to SARS-CoV-2 in house cats when human infection was spreading in the Lisbon area. Blood samples were collected from cats attending the Veterinary Teaching Hospital of the Faculty of Veterinary Medicine at the University of Lisbon (HEV-FMV-ULisboa). From these samples the seroprevalence of SARS-CoV-2 infection was estimated and virus neutralizing antibody activity was evaluated. For this purpose, we made use of enzyme-linked immunosorbent assay (ELISA) to identify sera with antibodies recognizing the Wuhan-Hu-1, Alpha, Delta, and Omicron variants of SARS-CoV-2. Afterwards, the positive samples were used in a seroneutralization solid phase assay to assess their activity in blocking SARS-CoV-2 receptor-binding domain (RBD) variants binding to the cognate ACE2 receptor. Finally, samples with highest blocking activity were tested in an infection neutralization assay using Wuhan-Hu-1 D614G, Delta, and Omicron variants of SARS-CoV-2 pseudotyped viral particles. In summary, this study provides valuable insights on the dynamics of human–cat SARS-CoV-2 transmission and on the development of effective and broadly neutralizing antibodies against SARS-CoV-2 in exposed cats.

## 2. Materials and Methods

### 2.1. Study Population

The study population consisted of 761 cats from the Lisbon region admitted to HEV-FMV-ULisboa. Among these animals, 733 were admitted to the hospital during the pandemic period (December 2019–October 2021) for several reasons, including vaccination, routine examinations, presurgical and specialty consultations, and infectious disease screening. While all 733 cats resided with owners during the SARS-CoV-2 2019–2021 outbreak period, information regarding owners' COVID-19 status was unavailable. This study also included 28 prepandemic samples collected between April 2018 and October 2019, used as negative controls in the different screenings.

### 2.2. Sample Collection, Processing, Storage, and Biobank Construction

Blood samples were collected by venipuncture into serum tubes with a clot accelerator and gel serum separator (Deltalab, Spain). The tubes were then centrifuged at 5000 × *g* for 5 min at room temperature (RT). Afterwards, the inert gel was placed between the clot and serum, enabling serum separation. Then, serum was centrifuged at 1600 × *g* for 10 min at 4°C, and a biobank was constructed and stored at −80°C. Sample collection was conducted after obtaining written pet owner consent according to EU recommendations for good practices and animal welfare. The study was approved by the Animal Care and Ethical Committee of the Faculty of Veterinary Medicine at the University of Lisbon (Ref No. 00142023).

### 2.3. Detection of Anti-SARS-CoV-2 Antibodies by ELISA

Cat serum samples were inactivated for 1 h at 56°C. Then, cat serum was screened for the presence of immunoglobulin M (IgM) and immunoglobulin G (IgG) against SARS-CoV-2. For the initial screening, a 96-well ELISA plate (Corning, USA) was coated with 50 µL/well of Spike–Wuhan-Hu-1 (0.5 µg/mL) and RBD–Wuhan-Hu-1 (2 µg/mL) proteins produced in house as described by Castro *et al*. [39]. The plate was washed with phosphate-buffered saline (PBS) supplemented with Tween20 0.1% (PanReac AppliChem ITW Reagents, USA) and nonspecific interactions were blocked with PBS supplemented with 2% bovine serum albumin (BSA; Merck, Germany) for 1 h at 37°C. After washing, serum samples were added at 1:50 dilution. A new washing step was performed and goat anti-cat IgM-horseradish peroxidase (HRP; ab112792, Abcam, UK) or goat anti-cat IgG-HRP (ab112801, Abcam, UK) were added at a 1:10,000 dilutions. One hour later, the washing step was repeated and 2,2′-azinobis (3-ethylbenzothiazoline-6-sulfonic acid) (ABTS; Merck, Germany) was added to the plate. Optical density (OD) at a wavelength of 415 nm was measured using an iMark Microplate Absorbance Reader (Bio-Rad Laboratories, USA). For each serum sample, OD values were measured for Spike, RBD, and BSA (control). The cutoff value for positivity was established at 0.5, which was calculated as twice the mean OD value of the BSA control. Serum samples exhibiting OD values for Spike and/or RBD proteins equal to or above than 0.5 were classified as positive. To evaluate how emerging RBD mutations affect antibody binding, a second ELISA screening was performed. For that, positive serum samples were further tested against different RBD variants of SARS-CoV-2 namely Alpha (B.1.1.7; abx620010, Abbexa, UK), Delta (B.1.617.2; abx620012, Abbexa, UK) or Omicron (B.1.1.529; abx620026, Abbexa, UK; 2 µg/mL), and the remaining steps of the protocol were followed as described above. The results were analyzed using GraphPad Prism version 8.0.1 (GraphPad Software, USA).

### 2.4. Surrogate Virus Neutralization Test (sVNT)

To detect neutralizing activity against the SARS-CoV-2 Wuhan-Hu-1 isolate, ELISA-positive samples were subjected to a commercial SARS-CoV-2 sVNT (GenScript, Netherlands), according to the manufacturer's instructions. This test detects the presence of neutralizing antibodies that bind specifically to the SARS-CoV-2 Wuhan-Hu-1 RBD domain and inhibit the interaction between the RBD and ACE2. A cutoff of 30% inhibition was established, as recommended by the manufacturer, for the sample to be considered positive. This assay was further repeated for Alpha (B.1.1.7), Delta (B.1.617.2), and Omicron (B.1.1.529) RBD SARS-CoV-2 variants (Z03595, Z03614, and Z03730; GenScript, Netherlands) for samples with positive result in ELISA against the respective variants.

### 2.5. Statistical Analysis

To evaluate whether there is a statistically significant difference in the binding levels of SARS-CoV-2 Wuhan-Hu-1 Spike and RBD between sVNT-positive and negative samples, we employed the Mann–Whitney *U* test due to the nonnormal distribution of the data. All analyses were conducted using Statistical Package for the Social Sciences (SPSS) version 29.0.0.0, with a significance level set at *p*  < 0.05.

### 2.6. Titration of Antibodies Against SARS-CoV-2

Serial dilutions (1:50, 1:100, 1:500, 1:1000, and 1:5000) were used to determine the antibody titer of the samples that tested positive by ELISA and sVNT assays. Sera at different dilutions were tested against Spike and RBD proteins of the SARS-CoV-2 Wuhan-Hu-1 isolate by ELISA performed as described above.

### 2.7. SARS-CoV-2 Pseudotyped-Based Neutralization Assays

The human embryonic kidney cell line HEK293T (CRL-1573, American Type Culture Collection (ATCC)) was maintained in Dulbecco's modified Eagle medium (DMEM) supplemented with 10% fetal bovine serum (FBS) (Gibco, USA) and 1% penicillin–streptomycin (Gibco, USA), and incubated at 37°C and 5% carbon dioxide (CO_2_). HEK293T cells were seeded at 8.5 × 10^5^ cells/well into six-well cell culture plates under the same conditions until they reached 60%–80% confluence. After 24 h, cells were transfected to produce lentiviral particles pseudotyped with the S protein of different variants of SARS-CoV-2 Wuhan-Hu-1 D614G (plv-cov2-sd19g, InvivoGen, USA), Delta variant (B.1.617.2; plv-spike-v8, InvivoGen, USA), and Omicron variant (B.1.1.529; plv-spike-v11, InvivoGen, USA). Transfection was performed using Lipofectamine 2000 reagent (Thermo Fisher Scientific, USA) in optimized minimal essential medium (Opti-MEM) medium (Gibco, USA) following the manufacturer's instructions. The plasmids used in the transfection process were composed of pLX311 luciferase (117735, Addgene, USA), the third generation Gag/Pol packaging plasmids pMDL (12251, Addgene) and pRSV/Rev (12253 Addgene) and the different SARS-CoV-2 viral envelopes. Approximately 4 h posttransfection, fresh DMEM containing 10% FBS and 1% penicillin–streptomycin was added to the cells, followed by incubation for 48 h at 37°C and 5% CO_2_. Supernatants were harvested, centrifuged at 2000 × *g* for 5 min at RT, and filtered through a 0.45 µm filter. The pseudovirus titer was quantified using the human immunodeficiency virus (HIV) p24 ELISA kit (Abcam, UK), according to the manufacturer's instructions.

HEK293T:ACE2 cells (hkb-hace2, InvivoGen, USA) were maintained in DMEM supplemented with 10% FBS (Gibco, USA), 1% penicillin–streptomycin (Gibco, USA) and 0.5 µg/mL puromycin (InvivoGen, USA) and incubated at 37°C and 5% CO_2_. HEK293T:ACE2 cells were seeded in 96- well cell culture plates at a density of 3 × 10^4^ cells/well under the same conditions. The SARS-CoV-2 pseudotyped viral particles were incubated for 1 h at 37°C and 5% CO_2_ with the selected pandemic serum samples at different dilutions (1:50, 1:100, 1:250, 1:500, 1:1000, 1:2000, 1:5000, 1:20,000, and 1:100,000). An irrelevant serum sample (#3201) collected during the pandemic period and without binding activity against SARS-CoV-2 Spike or RBD proteins or virus neutralization properties was included and used as a negative control. Mixtures of sera and pseudoviruses with a multiplicity of infection (MOI) of 10 were then added to the cells. Approximately 72 h after infection, cells were washed with PBS (Gibco, USA), lysed and luciferin substrate was added according to the manufacturer's instructions (Pierce Firefly Luciferase Glow Assay Kit, Thermo Fisher Scientific, USA). Sera neutralizing activity was characterized based on luciferase activity by measuring a bioluminescent signal using GloMax 96 Microplate Luminometer (Promega, USA). The neutralizing profile of each serum sample was obtained using GraphPad Prism version 8.0.1 through a nonlinear regression curve of log10 inhibitor dilution versus normalized response with a variable slope (four parameters).

## 3. Results

### 3.1. Detection of Serum Antibodies Against Spike and RBD SARS-CoV-2 Wuhan-Hu-1 Isolate

**Figure 1:**
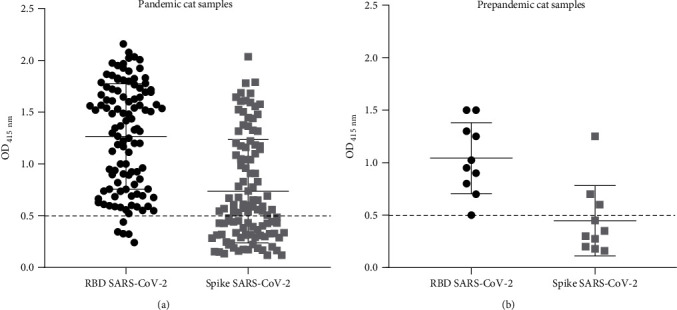
ELISA-positive pandemic and prepandemic cat samples against SARS-CoV-2 Spike and RBD proteins from Wuhan-Hu-1 isolate. A total of 761 cat serum samples (733 pandemic samples and 28 prepandemic samples from animals admitted to the HEV-FMV-ULisboa were subjected to ELISA against the spike and RBD proteins from SARS-CoV-2 Wuhan-Hu-1 isolate. (A) OD values of 108 ELISA-positive samples out of the 733 pandemic cat samples tested. (B) OD values of 10 ELISA-positive samples out of the 28 prepandemic cat samples tested. The cutoff value for positivity was established at 0.5, which was calculated as twice the mean OD value of the BSA control. Serum samples exhibiting OD values for Spike and/or RBD proteins equal to or above than 0.5 were classified as positive. BSA, bovine serum albumin; CoV-2, coronavirus 2; ELISA, enzyme-linked immunosorbent assay; OD, optical density; RBD, receptor-binding domain; SARS, severe acute respiratory syndrome.

The study population comprised a total of 761 cats, with 733 admitted to the HEV-FMV-ULisboa during the pandemic period (December 2019–October 2021), while the remaining 28 cats were admitted during the prepandemic period (April 2018–October 2019) and used as a negative control in the screenings. First, all the samples were tested for the presence of IgM and IgG antibodies against SAR-CoV-2. For this, an initial ELISA screening targeting the spike and RBD proteins from the SARS-CoV-2 Wuhan-Hu-1 isolate was performed. Samples were considered positive if their OD at 415 nm equaled or were above the predetermined cutoff value for at least one of the antigens (spike or RBD). The results showed no IgM antibodies against either of the two SARS-CoV-2 antigens (data not shown). In contrast, 14.7% (108/733) of the pandemic samples tested positive for IgG antibodies against at least one of the two SARS-CoV-2 antigens (Figure 1A). Regarding the analysis of prepandemic samples, surprisingly, some serum samples (10/28) recognized SARS-CoV-2 proteins, mainly the RBD domain. The prepandemic samples essentially recognized the Wuhan-Hu-1 RBD protein (10/10), although some samples (3/10) recognized the Wuahn-Hu-1 Spike protein (Figure 1B). A total of 118 ELISA-positive samples (108 pandemic samples and 10 prepandemic samples) were subsequently tested using a sVNT to assess the presence of neutralizing antibodies against the Wuhan-Hu-1 isolate.

### 3.2. sVNT for the Detection of Neutralizing Antibodies Against SARS-CoV-2 Wuhan-Hu-1 Isolate

**Figure 2:**
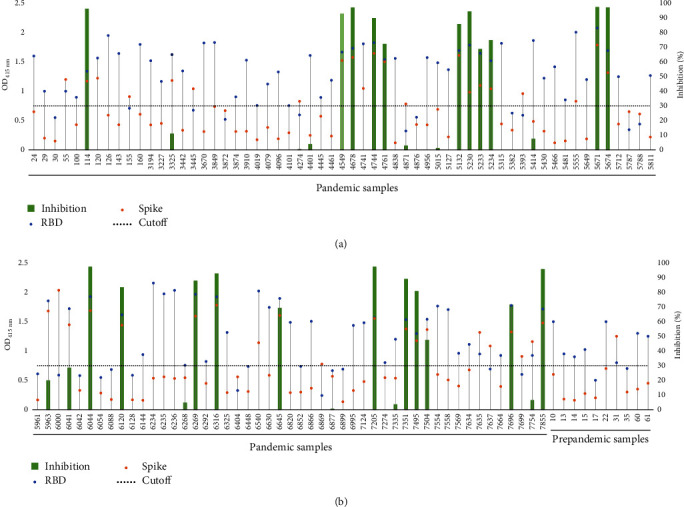
Binding activity versus RBD:ACE2 inhibition percentages obtained in the sVNT assay with RBD Wuhan-Hu-1 for ELISA-positive samples. Considering a cutoff of 30% for RBD:ACE2 inhibition were identified 20.4% (22/108) positive samples. The *Y*-axis on the left represents the different ODs at 415 nm obtained for RBD (blue) and Spike (orange) proteins in ELISA for the different positive samples. The *Y*-axis on the right shows the % inhibition of RBD:ACE2 binding in the sVNT assay, with the green bars corresponding to the values obtained for each sample. (A) Pandemic samples (24 to 5811). (B) Pandemic samples (5961 to 7855) and prepandemic samples (10-61). ACE2, angiotensin-converting enzyme II; ELISA, enzyme-linked immunosorbent assay; OD, optical density; RBD, receptor-binding domain; sVNT, surrogate virus neutralization test.

**Figure 3:**
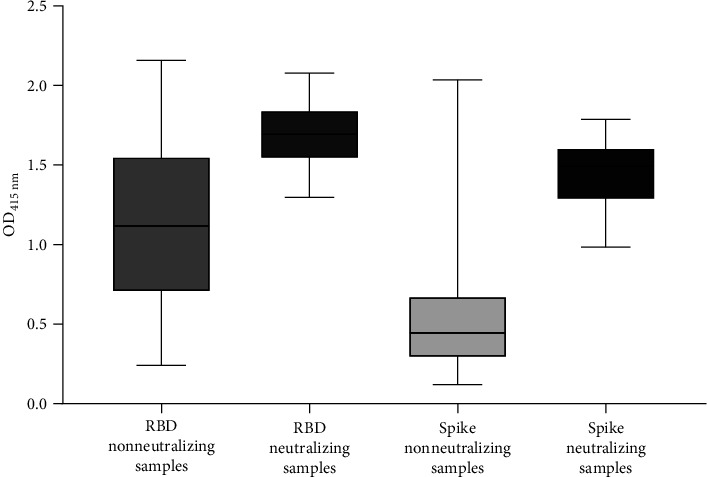
Range of OD values obtained by ELISA for SARS-CoV-2 Wuhan-Hu-1 RBD and Spike proteins and data crossing with sVNTresults. Crossing the results of the sVNT and ELISA, the samples were divided into four groups: RBD nonneutralizing samples, RBD neutralizing samples, Spike nonneutralizing samples, and Spike neutralizing samples. sVNT-positive samples demonstrated a significantly higher level of binding against the SARS-CoV-2 Wuhan-Hu-1 Spike and RBD by ELISA compared to sVNT-negative samples (*p* < 0.05, Mann–Whitney *U* test). CoV-2, coronavirus 2; ELISA, enzyme-linked immunosorbent assay; SARS, severe acute respiratory syndrome; OD, optical density; RBD, receptor-binding domain; sVNT, surrogate virus neutralization test.

ELISA-positive pandemic and prepandemic samples were subjected to a commercial sVNT assay to detect neutralizing activity against the SARS-CoV-2 Wuhan-Hu-1 isolate. This test detects the presence of neutralizing antibodies that recognize the SARS-CoV-2 RBD domain and inhibit the binding of the RBD to solid phase ACE2. Among the 108 ELISA-positive pandemic samples subjected to the test, 20.4% (22/108) contained antibodies with RBD-ACE2 blocking properties (Figures 2A, B). In contrast, the 10 ELISA-positive prepandemic samples were negative in the sVNT assay (Figure 2B). Our results revealed an interesting observation, sVNT-positive samples demonstrated a significantly higher level of binding against the SARS-CoV-2 Wuhan-Hu-1 Spike and RBD by ELISA compared to sVNT-negative samples (*p* < 0.05, Mann–Whitney *U* test; Figure 3).

Regarding the prepandemic samples, it is important to note that all of them tested positive for antibodies against feline coronavirus (FCoV) using the FCoV-ELISA Cat (Afosa) test. These binding profiles and cross-reactivity between SARS-CoV-2 and FCoV in prepandemic samples have also been observed and reported by other groups [40–42]. Since no neutralizing antibodies against SARS-CoV-2 were detected in the prepandemic samples and we verified cross-reactivity against the FCoV virus, these samples were not subjected to further assays. Moreover, to evaluate possible cross-reactivity with FCoV, the 22 sVNT-positive pandemic samples were screened for antibodies against the FCoV using the same test mentioned above. Except for one sample (5671), which could not be tested due to insufficient serum, all samples tested negative (data not shown). Therefore, only the 22 sVNT-positive pandemic samples were used in the subsequent assays for further characterization.

### 3.3. Titration of Antibodies Against SARS-CoV-2 Wuhan-Hu-1 Isolate

**Figure 4:**
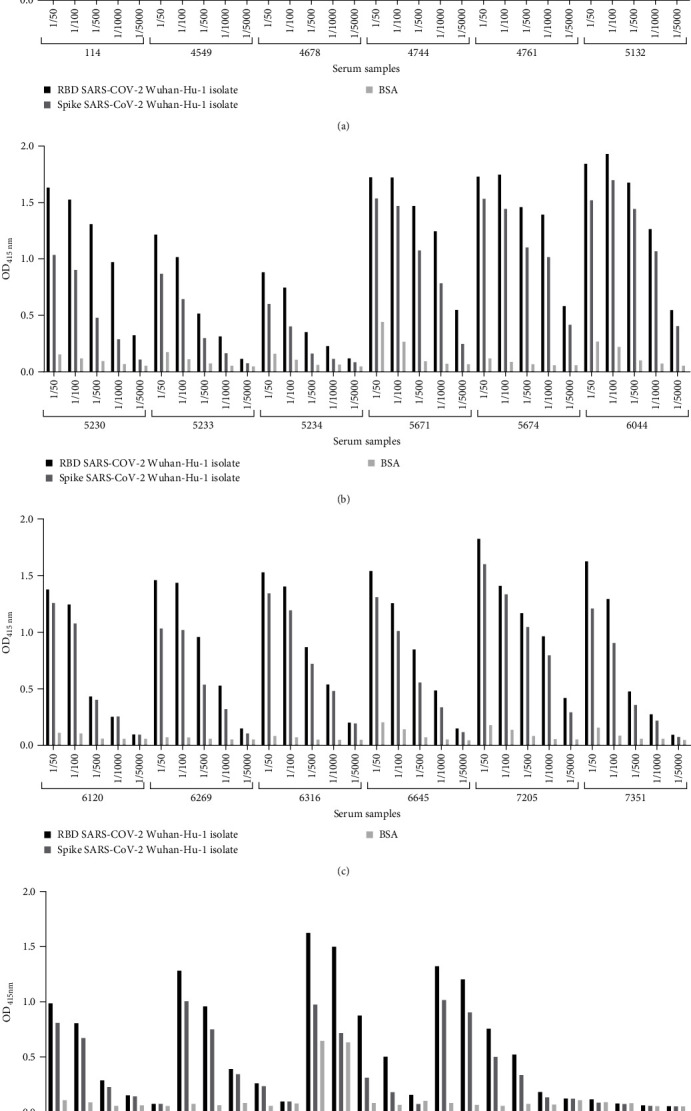
Titration of antibodies against SARS-CoV-2 Wuhan-Hu-1 spike and RBD proteins in the 22 most promising pandemic samples. Based on ELISA and sVNT assays performed against the SARS-CoV-2 Wuhan-Hu-1 isolate, 22 positive samples were identified and titrated. Among these 22 samples, a titer of 1:1000 was obtained in 17 samples, and a titer of 1:5000 was obtained in five samples (114, 5671, 5674, 6044, and 7205). The negative control was a serum sample collected during the pandemic period and without binding activity against SARS-CoV-2 Spike or RBD proteins or sVNT virus neutralization properties. (A) From sample 114 to 5132, (B) from sample 5230 to 6044, (C) from sample 6120 to 7351, and (D) from sample 7495 to 7855. CoV-2, coronavirus 2; ELISA, enzyme-linked immunosorbent assay; RBD, receptor-binding domain; SARS, severe acute respiratory syndrome; sVNT, surrogate virus neutralization test.

To further characterize the 22 sVNT-positive pandemic samples, antibody titers were determined by ELISA against spike and RBD proteins of the SARS-CoV-2 Wuhan-Hu-1 isolate. Of the 22 samples analyzed, 17 showed an antibody titer of 1:1000 and five showed an antibody titer of 1:5000 (samples 114, 5671, 5674, 6044, and 7205). The titrations of different samples are shown in Figure 4. Analysis of the titration results showed that the samples with the highest antibody titer were coincident with the samples with the highest signal in the previous ELISA and the highest percentage of inhibition of the RBD:ACE2 interaction in the sVNT assay (Figure 2).

### 3.4. Detection of Antibodies Capable of Recognizing and Neutralizing SARS-CoV-2 Variants of Concern (VOCs)

**Figure 5:**
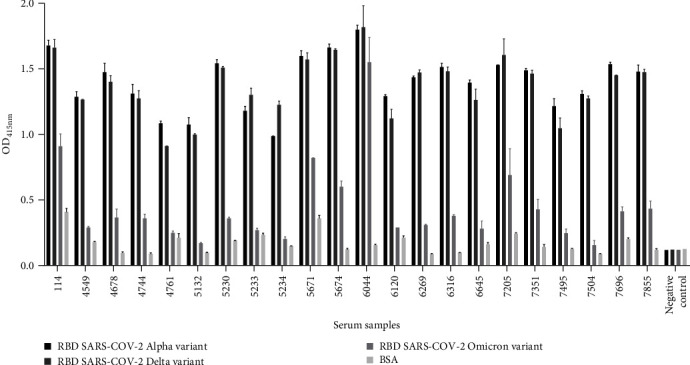
Comparative ELISA analysis of the 22 most promising samples for SARS-CoV-2 specific antibodies against the VOCs (Alpha, Delta, and Omicron). The 22 most promising serum samples were subjected to ELISA to evaluate the presence of antibodies capable of recognizing Alpha, Delta, and Omicron variants. All 22 serum samples demonstrated high binding to SARS-CoV-2 Alpha and Delta variants. Regarding the Omicron variant, samples 114, 5671, 5674, 6044, and 7205 were the samples with the highest binding against this variant. The negative control was a serum sample collected during the pandemic period and without binding activity against SARS-CoV-2 Spike or RBD proteins or sVNT virus neutralization properties. CoV-2, coronavirus 2; ELISA, enzyme-linked immunosorbent assay; RBD, receptor-binding domain; SARS, severe acute respiratory syndrome; sVNT, surrogate virus neutralization test; VOCs, variants of concerns.

**Figure 6:**
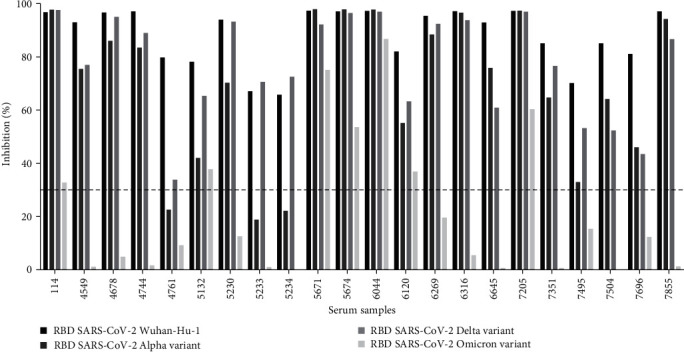
sVNT results against SARS-CoV-2 Wuhan-Hu-1, Alpha, Delta, and Omicron variants. The 22 most promising serum sampleswere subjected to sVNT to evaluate the presence of antibodies capable of neutralizing different VOCs. All 22 samples presented neutralizing antibodies against Wuhan-Hu-1 isolate and Delta variants. Regarding the Alpha variant, all samples tested positive for neutralizing antibodies against this variant except 4761, 5233, and 5234. Despite having antibodies with neutralizing properties, samples 4761, 5233, and 5234 failed to reach the 30% cutoff established to be considered positive. Samples 114, 5132, 5671, 5674, 6120, 6044, and 7205 were the only ones with neutralizing antibodies against the Omicron variant. The dashed black line represents the 30% cutoff. CoV-2, coronavirus 2; SARS, severe acute respiratory syndrome; sVNT, surrogate virus neutralization test; VOCs, variants of concerns.

After estimating the antibody titers against the Wuhan-Hu-1 isolate in the 22 sVNT-positive pandemic samples, these samples were subjected to further testing. Thus, an ELISA was conducted to determine the presence of antibodies against the RBD of three VOCs: Alpha, Delta, and Omicron (Figure 5). All samples exhibited strong binding activity against Alpha and Delta RBD variants. However, when binding against the Omicron RBD variant was analyzed, only five serum samples (114, 5671, 5674, 6044, and 7205) showed significant binding for this variant. Importantly, these five samples were the only ones that demonstrated an antibody titter of 1:5000, which was higher than that of the remaining samples tested (1:1000; Figure 4). Next, we tested whether antibodies recognizing SARS-CoV-2 VOCs exhibited neutralizing properties. Thus, the 22 positive samples were subsequently analyzed in an sVNT to detect the presence of neutralizing antibodies against the Alpha, Delta, and Omicron variants. All the samples tested positive for sVNT against the SARS-CoV-2 Wuhan-Hu-1 isolate and Delta variant. Regarding the Alpha variant, most of the samples showed a positive result, with the exception of samples 4761, 5233, and 5234. Despite these samples presented antibodies with slight neutralizing properties for this variant, it was not enough to reach the 30% cutoff established to be considered positive. Regarding the Omicron variant, the same five samples (114, 5671, 5674, 6044, and 7205) highlighted in the previous ELISA assay showed a strongest ability to inhibit the RBD:ACE2 interaction for this variant (Figure 6). In addition, despite not having been highlighted by the highest binding to the Omicron variant in the ELISA assay, samples 5132 and 6120 also presented antibodies with neutralizing properties against this variant in the sVNT.  Table 1 summarizes the data obtained for the 22 sVNT-positive pandemic serum samples with RBD-ACE2 blocking activity, and antibody titer, including gender, age, and the sample collection date.

### 3.5. Assessing Cat Antibodies Neutralizing Potential Against SARS-CoV-2 Pseudotyped Viruses

**Figure 7:**
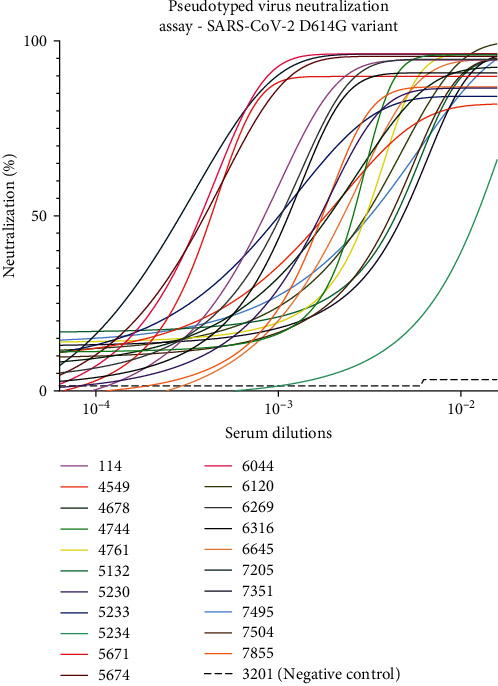
Pseudotyped virus neutralization assay conducted against the SARS-CoV-2 Wuhan-Hu-1 D614G variant. This assay evaluatedthe neutralizing potential of the 22 most promising serum samples in inhibiting the infection process by the SARS-CoV-2 Wuhan-Hu-1 D614G variant. Among these, serum samples 114, 5671, 5674, 6044, and 7205 exhibited the most promising outcomes in ELISA and sVNT for any of the variants tested. Notably, in the pseudotyped virus assays against the Wuhan-Hu-1 D614G variant, these five serum samples demonstrated ahigher ability to neutralize the infection, even at higher dilutions. No neutralization was observed in the negative control serum (sample 3201) asample collected during the pandemic period and without binding activity against SARS-CoV-2 Spike or RBD proteins or sVNT virus neutralization properties. Sample 7696 was not included in the assay due to lack of serum. CoV-2, coronavirus 2; ELISA, enzyme-linked immunosorbent assay; RBD, receptor-binding domain; SARS, severe acute respiratory syndrome; sVNT, surrogate virus neutralization test.

**Figure 8:**
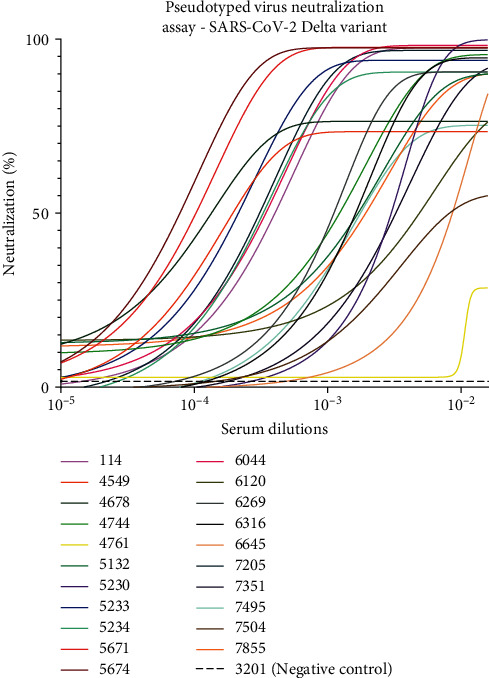
Pseudotyped virus neutralization assay conducted against the SARS-CoV-2 Delta variant. This assay evaluated the neutralizingpotential of the 22 most promising serum samples in inhibiting the infection process by the SARS-CoV-2 Delta variant. Among these, serum samples114, 5671, 5674, 6044, and 7205 exhibited the most promising outcomes in ELISA and sVNT for any of the variants tested. Notably, in the pseudotyped virus assays against the SARS-CoV-2 Delta variant, these five serum samples demonstrated a higher ability to neutralize the infection, even at higher dilutions. No neutralization was observed in the negative control serum (sample 3201) a sample collected during the pandemic period and without binding activity against SARS-CoV-2 Spike or RBD proteins or sVNT virus neutralization properties. Sample 7696 was not included in the assay due to lack of serum. CoV-2, coronavirus 2; ELISA, enzyme-linked immunosorbent assay; RBD, receptor-binding domain; SARS, severe acute respiratory syndrome; sVNT, surrogate virus neutralization test.

**Figure 9:**
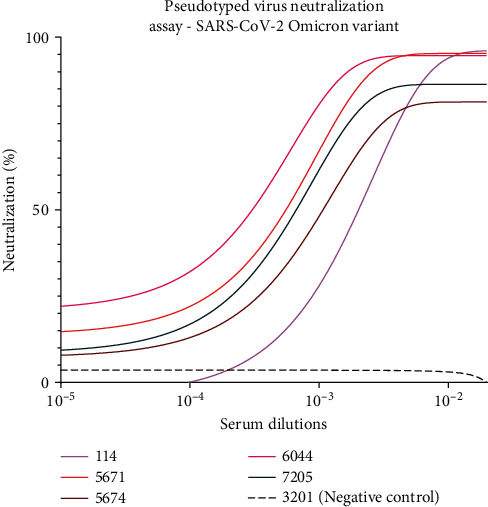
Pseudotyped virus neutralization assay conducted against the SARS-CoV-2 Omicron variant. This assay evaluated the neutralizing potential of the five most promising serum samples in inhibiting the infection process by the SARS-CoV-2 Omicron variant. Serum samples 114, 5671,5674, 6044, and 7205 exhibited the most promising outcomes in ELISA and sVNT for any of the variants tested. Notably, in the pseudotyped virus assays against the Omicron variant, these five serum samples demonstrated a higher ability to neutralize the infection, even at higher dilutions. Noneutralization was observed in the negative control serum (sample 3201) a sample collected during the pandemic period and without binding activity against SARS-CoV-2 Spike or RBD proteins or sVNT virus neutralization properties. CoV-2, coronavirus 2; ELISA, enzyme-linked immunosorbent assay; RBD, receptor-binding domain; SARS, severe acute respiratory syndrome; sVNT, surrogate virus neutralization test.

To determine the neutralization activity of the 22 sVNT-positive pandemic samples with RBD- ACE2 blocking properties to prevent infection, we generated lentiviral particles pseudotyped with the spike proteins of the SARS-CoV-2 Wuhan-Hu-1 D614G, Delta (B.1.617.2), and Omicron (B.1.1.529) variants. SARS-CoV-2 pseudotyped lentiviral particles were assembled by transfecting HEK293T cells and after harvested and tittered were used to infect HEK293T:ACE2 cells. The infection process was monitored using the luciferase reporter gene carried by the virus particles allowing the emission of a bioluminescent signal by the infected cells upon incubation with luciferin. As shown in Figures 7 and 8, samples that presented RBD-ACE2 blocking activity in the sVNT assay effectively neutralized infection by the Wuhan-Hu-1 D614G and Delta variants of SARS-CoV-2 pseudotyped viral particles. In contrast, when an irrelevant serum (#3201) with nonbinding properties against SARS-CoV-2 was used as a negative control, no neutralization activity was observed. Notably, samples 114, 5671, 5674, 6044, and 7205 exhibited higher neutralization properties even at higher dilutions. These serum samples displayed strong binding activity and effective neutralization properties in ELISA and sVNT assays, respectively, with the highest antibody titers. Moreover, samples 114, 5671, 5674, 6044, and 7205, which presented the best results in ELISA and sVNT against the SARS-CoV-2 Omicron variant, effectively blocked the infection process when tested against this variant with pseudotyped virus (Figure 9).

### 3.6. Discussion and Conclusion

Since the onset of the pandemic, SARS-CoV-2 infection have been reported in several animal species [5, 7]. Among these, cats pose a significant concern due to their proximity to humans and other domesticated animals. Seroprevalence studies have proven to be valuable tools for identifying previous exposure to the virus, serving as a good indicator of the actual prevalence of SARS-CoV-2 in cats. Within this context, in the present study we aimed to evaluate the exposure and antibody response to SARS-CoV-2 in house cats when human infection was spreading in the Lisbon area. For this purpose, the seroprevalence of SARS-CoV-2 in these animals was assessed and the potential of antibodies to neutralize the virus was evaluated. Samples from 761 cats admitted to the HEV-FMV-ULisboa were collected and characterized (733 pandemic samples and 28 prepandemic samples). An in-house ELISA was established to screen for the presence of IgM and IgG antibodies against SARS-CoV-2 Spike and RBD proteins from the Wuhan-Hu-1 isolate. From a total of 733 pandemic samples tested, 14.7% (108/733) were identified as IgG-positive based on the recognition of these proteins. Unexpectedly, among the 28 prepandemic samples analyzed, 10 exhibited positive results for anti-SARS-CoV-2 IgG antibodies by ELISA. ELISA-positive pandemic and prepandemic samples were further examined using an sVNT assay to detect neutralizing antibodies against the SARS-CoV-2 Wuhan-Hu-1 isolate. Among the pandemic samples, 20.4% (22/108) exhibited neutralizing antibodies. Notably, none of the 10 prepandemic samples with positive result in ELISA exhibited detectable neutralizing antibodies against SARS-CoV-2 in the sVNT assay. Since in these prepandemic samples no neutralizing antibodies against SARS-CoV-2 were detected these samples did not proceed into further assays against other variants of SARS-CoV-2. Based on these results, the 22 most promising pandemic samples were retested using ELISA and sVNT against Alpha, Delta, and Omicron SARS-CoV-2 variants. Our data demonstrated that these samples contained antibodies capable of recognizing and neutralizing these variants. Moreover, the five most promising samples (114, 5671, 5674, 6044, and 7205) exhibited superior neutralizing capabilities, even at higher dilutions and inclusive against the Omicron variant. Thus, despite being collected before detection of the Omicron variant in the human population, these five samples presented antibodies that recognized and neutralized this variant [16, 43].

Similar to other studies, our data demonstrated the exposure of cats to SARS-CoV-2. However, even in seroprevalence studies, variable estimates can be found depending on the methodology used for antibody detection, screening, and cutoff implemented. For example, a study in a population of cats from Germany, the United Kingdom, Italy, and Spain conducted by Schulz et al. [44] estimated a SARS-CoV-2 specific antibodies seroprevalence of 4.3% (92/2160) and 4.4% (96/2160) using a RBD-ELISA or sVNT, respectively. In another study, Jara et al. [45] used sVNT assays and identified 17.1% (7/41) and 31.7% (13/41) of cat-positive samples using cutoff inhibition values of 30% and 20%, respectively. Another survey performed on a population of cats in Wuhan, 14.7% (15/102) of the collected serum samples were positive by RBD-ELISA, and 11 of these samples had SARS-CoV-2 neutralizing antibodies with titers ranging from 1:20 to 1:1080 [46]. A survey conducted by Barroso et al. [19] and performed in five districts of Portugal (Braga, Porto, Aveiro, Coimbra, and Lisbon) also used ELISA assays against SARS-CoV-2 RBD and reported a seroprevalence of 21.74%, which was similar to that estimated in our study. In Portugal, another study carried out in a population of cats found a seroprevalence of 5.0% in COVID-19 positive households and 0.7% in COVID-19 negative households [47]. However, in this study, antibody screening was performed against the SARS-CoV-2 nucleocapsid protein and as already mentioned by the authors, this may be the reason for the lower seroprevalence observed in this study. Also in our study, when all samples were tested by ELISA for IgG against SARS-CoV-2 Wuhan-Hu-1 isolate, 14.7% (108/733) of samples tested positive. Then, ELISA-positive samples were subjected to a sVNT against the same isolate, and 20.4% (22/108) of the samples harbored neutralizing antibodies against the virus. Crossing the results of both tests suggests that only 3% (22/733) of the animals were exposed to SARS-CoV-2 and generated neutralizing antibodies. However, it remains unclear whether the detection of antibodies against the spike and RBD can be an indication of exposure to SARS-CoV-2 or whether only the detection of neutralizing antibodies can provide this guarantee. Thus, considering our results as well as this discrepancy in the reported results, it is important to standardize the types of screening and cutoff values to improve comparisons between studies and the seroprevalence percentages obtained.

Seroprevalence studies reported on cats have indicated that these animals may be infected by their owners. In fact, when studies were conducted in groups of animals whose owners tested positive for SARS-CoV-2, the reported seroprevalence rates were higher. In a survey performed by Fritz et al. [21] on a population of household cats and dogs whose owners had laboratory-confirmed SARS-CoV-2 infections, the seroprevalence of SARS-CoV-2 antibodies ranged from 21% to 53%. In another study, Alberto-Orlando et al. [48] estimated a seroprevalence of 12.5% in cats and 26.2% in dogs in both groups of samples from households where SARS-CoV-2 infection was confirmed.

Another point highlighted by our study is the cross-reactivity between SARS-CoV-2 and other coronaviruses. We used 28 prepandemic samples, 10 of which tested positive for anti-SARS-CoV-2 antibodies by ELISA. However, none of the 10 samples tested positive for neutralizing antibodies against SARS-CoV-2 in the sVNT assay and all samples tested positive for FCoV. Cross-reactivity between SARS-CoV-2 and FCoV has also been evaluated in other studies [40–42]. In a survey conducted by Yilmaz et al. [40] with a methodology very similar to ours, six prepandemic serum samples were positive in the RBD-ELISA; none of the samples tested positive in the sVNT assay and 5/6 samples tested positive for antibodies against FCoV. Yamamoto et al. [41] also highlights the existence of cross-reactive antibodies to SARS-CoV-2 RBD in the sera of laboratory cats infected with well-characterized FCoV1 and FCoV2 strains from pre-COVID-19 pandemic years. Furthermore, the alignment analyses of SARS-CoV-2 RBD and strains from FCoV serotypes 1 and 2 demonstrated an amino acid sequence identity and similarity of 11.5% and 31.8% with FCoV1 RBD, respectively, and 12.2% identity and 36.5% similarity for FCoV2 RBD [41]. In March 2022, another study was published with prepandemic feline serum samples, of which approximately 50% tested positive for anti-SARS-CoV-2 RBD antibodies and some tested negative for FCoV. With the appearance of positive prepandemic samples in the SARS-CoV-2 RBD-ELISA, but negative for FCoV, this publication raises the possibility that another agent, already in circulation before the pandemic, has generated this cross-reactivity with SARS-CoV-2 [42].

Another concern is whether cats infected with SARS-CoV-2 can transmit the virus to humans. To date, spillback events have been reported in minks, hamsters, cats, and white-tailed deer [49–52]. In cats, human-to-cat and cat-to-cat transmissions are the most frequently reported [17, 20, 24, 53]. Regarding cat-to-human transmission, only one study was reported [51]. In this case, SARS-CoV-2 infected owners transmitted the virus to their cats, and one of the animals in turn transmitted the virus to the veterinarian who assisted him [51]. Hence, although spillback events are rare, they should be considered. The possibility of natural or experimental infection with SARS-CoV-2 has been demonstrated in several species. Thus, it is imperative that these species be monitored to assess whether they may constitute intermediate hosts, viral reservoirs, and/or whether they may play an active role in virus transmission. By serving as intermediate hosts or viral reservoirs, these species may pose risks not only to humans, but also to other animal species. Not identifying which species are susceptible to infection allows the virus to adapt and evolve in different hosts, leading to an increase of new viral variants and possibly new species susceptible to human infection [43]. Furthermore, the evolution of the virus in these animals and the appearance of new variants could also be decisive in the loss of effectiveness of currently available vaccines, which compromise the owners of these animals as well as the human population in general. Implementing a “One Health” approach is crucial to mitigate the spread of SARS-CoV-2 in animals and humans, anticipate the detection of new agents, and prevent its spread through zoonotic events.

Although cats do not seem to play an important role in the active dissemination of the virus, it is essential to understand the transmission dynamics driving SARS-CoV-2 infection in these animals. An important step would be to assess which owner-cat behavior/interactions constitute potential risk factors for promoting viral transmission. As such, some studies have evaluated whether proximity and maintenance of owner-animal interactions constitute risk factors that may be associated with human–animal spillover. A study of domestic dogs and cats showed that when owners were diagnosed with COVID-19 and avoided contact with their pets, none of the pets seroconverted. However, when this contact was maintained, 21% of the animals became seropositive [54]. Sharing beds or food with an infected owner or contracting a new disease are some of the risk factors already identified to be associated with pet seropositivity [48, 55]. Until sufficient information is made available on this point, a good practice would be to limit contact between the animals and persons diagnosed with COVID-19. Due to the importance of these studies, we are currently conducting another study on cats from households whose owners have been infected with SARS-CoV-2 to investigate whether there is a significant change in the seroprevalence of SARS-CoV-2 in these animals. Additionally, we are also attempting to identify the potential risk factors that might be involved in human-to-cat transmission dynamics.

In summary, we successfully characterized the humoral immune response in house cats exposed to SARS-CoV-2 when human infection was spreading in the Lisbon area. Based on our results, we conclude that cats are susceptible to SARS-CoV-2 infection and develop highly potent and broadly neutralizing antibodies against different virus variants. Importantly, some of these animals developed antibodies capable of neutralizing the Omicron variant even before this variant was in circulation. Therefore, we believe that cats naturally infected with SARS-CoV-2 represent a valuable anthroponotic disease model in house settings and might be a promising source to develop recombinant antibodies against the virus. In this context, we are currently constructing antibody single-chain variable fragment (scFv) phage libraries from peripheral blood mononuclear cells (PBMC) isolated from cats naturally infected with SARS-CoV-2, to develop a new generation of neutralizing antibodies for COVID-19 and for future outbreaks. Thus, we believe that characterizing the immune response to SARS-CoV-2 in cats can contribute to the development of new preventive or therapeutic strategies, not only for humans but also for a broader spectrum of susceptible species, such as feline populations.

## Figures and Tables

**Table 1:** Summarizes the data obtained for the 22 sVNT-positive pandemic serum samples with RBD-ACE2 blocking activity.

Sample	Gender	Age (years)	Collection date	ELISA				sVNT				Antibody titer
				Wuhan-Hu-1	Alpha	Delta	Omicron	Wuhan-Hu-1	Alpha	Delta	Omicron	
114	M	N/A	07.05.2021	+	++	++	+	Y	Y	Y	Y	1:5000
4549	M	2	01.07.2021	++	+	+	Low	Y	Y	Y	N	1:1000
4678	F	4	01.07.2021	++	+	+	Low	Y	Y	Y	N	1:1000
4744	F	7	30.06.2021	++	+	+	Low	Y	Y	Y	N	1:1000
4761	M	15	30.06.2021	++	+	+	Low	Y	N	Y	N	1:1000
5132	F	2	19.07.2021	++	+	+	Low	Y	Y	Y	Y	1:1000
5230	M	12	22.07.2021	++	++	++	Low	Y	Y	Y	N	1:1000
5233	F	8	22.07.2021	++	+	+	Low	Y	N	Y	N	1:1000
5234	M	8	22.07.2021	++	+	+	Low	Y	N	Y	N	1:1000
5671	F	12	10.08.2021	++	++	++	+	Y	Y	Y	Y	1:5000
5674	F	1	13.08.2021	++	++	++	+	Y	Y	Y	Y	1:5000
6044	M	14	06.09.2021	++	++	++	++	Y	Y	Y	Y	1:5000
6120	M	8	06.09.2021	++	+	+	Low	Y	Y	Y	Y	1:1000
6269	F	N/A	06.09.2021	++	+	+	Low	Y	Y	Y	N	1:1000
6316	M	3	14.09.2021	++	++	+	Low	Y	Y	Y	N	1:1000
6645	M	8	16.09.2021	++	+	+	Low	Y	Y	Y	N	1:1000
7205	F	3	06.10.2021	++	++	++	+	Y	Y	Y	Y	1:5000
7351	M	12	12.10.2021	++	+	+	Low	Y	Y	Y	N	1:1000
7495	F	4	27.10.2021	+	+	+	Low	Y	Y	Y	N	1:1000
7504	M	13	15.10.2021	++	+	+	Low	Y	Y	Y	N	1:1000
7696	F	1	25.10.2021	++	++	+	Low	Y	Y	Y	N	1:1000
7855	M	2	26.10.2021	++	++	+	Low	Y	Y	Y	N	1:1000

*Note:* Each sample is followed by the respective information including animal's gender, age, collection date, and results in ELISA, sVNT, and antibody titer.

Abbreviations: +, 0.5 < OD_415 nm_ ≤ 1.5; ++, 1.5 < OD_415 nm_ ≤ 2.0; ACE2, angiotensin-converting enzyme II; ELISA, enzyme-linked immunosorbent assay; F, female; Low: OD_415 nm_ ≤ 0.5; M, male; N, inhibition percentage < 30%; N/A, not available; OD, optical density; RBD, receptor-binding domain; sVNT, surrogate virus neutralization test; Y, inhibition percentage ≥ 30%.

## Data Availability

The data used to support the findings of this study are included in the article.
